# Colorado and the Eastern Pulmonary Case

**Published:** 1908-01

**Authors:** 


### COLORADO AND THE EASTERN PULMONARY CASE.

---

The above is the title of a paper read before the meeting of the Mississippi Valley Medical Association at Columbus, Ohio, by Dr. W. A. Campbell, of Colorado Springs, Colo. The text for the paper was an analysis of four hundred cases sent to Colorado for lung troubles. They were recent arrivals and illustrate very well the character of the cases going to Colorado for health purposes.

Of this number, 32 per cent were in the incipient stage of the disease; 38 per cent moderately advanced in the disease; and 30 per cent were far advanced in the disease. Of the incipient cases, 91 per cent were improved; of the moderately advanced, 62 per cent were improved; while in the far advanced, 25 per cent improved for a time and life made more tolerable. Of the whole number, 19 per cent, and of the incipient cases, 65 per cent were arrested or cured. 68 per cent of the cases came from families having a tubercular history; of this number, 58 per cent improved. The improvement in the non-tubercular families was 62 per cent. Those with hemorrhages showed 58 per cent improved, while the non-hemorrhagic cases showed 64 per cent improved. The left lung was affected alone in 25 per cent of the cases. The right lung alone in 36 percent, and both lungs in 39 per cent.

He insists on the early detection of the disease and early protection of the patient by intelligent home treatment, sanitarium treatment or climatic treatment. The early detection of the disease rests with the family physician. It is he who must be taught to recognize the disease in its incipiency, if we are to get the best results from treatment. He recognizes only the specifics nutritious food, supervision of the patient and the open air. He speaks of the good work done by many in the home by enlightening the patients and properly directing his movements. He thinks these things are better carried out in a properly regulated sanitarium, and still better when a climate is selected that is adapted to the patients requirements.

He would not advise those with excessive temperatures, high pulse rate and extensive infiltration of the lungs to go to a high altitude until the acute symptoms have subsided. Acute miliary tuberculosis, or those with extensive softening, do not do well. Acute heart lesions should not go, but chronic cases where compensation is well established, may go without ill effects. Those with advanced chronic nephritis should not be sent to the high altitude. Advanced fibroid and emphysematous cases are not comfortable. Hemorrhagic cases do well if the hemorrhage occur early in the disease. One of the most important conditions is that the patient have sufficient finances to keep him until improvement is well established.

He insists that every case should be well studied before being sent to Colorado; all cases do not do well. The cause for failure to improve more often lays with the family physician, who fails to make an early diagnosis and give good advice, than it does with the climate of the locality selected for the patient. Colorado climate, with proper supervision of the patient, will arrest more than 90 per cent of the incipient cases of pulmonary tuberculosis.

(Author's abstract.)

